# *DHCR7* rs12785878 T>C Polymorphism Is Associated With an Increased Risk of Early Onset of Alzheimer's Disease in Chinese Population

**DOI:** 10.3389/fgene.2021.583695

**Published:** 2021-02-22

**Authors:** Xixi Liu, Pengfei Wu, Lu Shen, Bin Jiao, Xinxin Liao, Haochen Wang, Jiangnan Peng, Zhangyuan Lin

**Affiliations:** ^1^Department of Neurology, Xiangya Hospital, Central South University, Changsha, China; ^2^Hunan Provincial Key Laboratory of Medical Genetics, School of Life Sciences, Central South University, Changsha, China; ^3^Key Laboratory of Hunan Province in Neurodegenerative Disorders, Central South University, Changsha, China; ^4^National Clinical Research Center for Geriatric Diseases, Changsha, China; ^5^Key Laboratory of Organ Injury, Aging and Regenerative Medicine of Hunan Province, Changsha, China; ^6^Department of Geriatrics Neurology, Xiangya Hospital, Central South University, Changsha, China; ^7^Department of Orthopedics, Xiangya Hospital, Central South University, Changsha, China

**Keywords:** vitamin D insufficiency, Alzheimer's disease, single neucleotide polymorphism, genetic association analysis, 25(OH)D

## Abstract

**Background:** Vitamin D insufficiency has been considered a risk factor for Alzheimer's disease (AD) in several studies. Recently, four single-nucleotide polymorphisms (SNPs) to be genome-wide significant for 25-hydroxyvitamin D [25(OH)D] were identified to have an association with the risk of AD. These include *GC* rs2282679 A>C, *CYP2R1* rs10741657 T>C, *DHCR7* rs12785878 T>C, and *CYP24A1* rs6013897 T>A. However, the association between these polymorphisms and AD susceptibility in the Chinese population remains unclear.

**Methods:** A case-control cohort study was conducted in 676 AD patients (mean age at onset was 69.52 ± 10.90 years, male: 39.2%) and 551 healthy controls (mean age was 67.73 ± 6.02 years, male: 44.8%). Genotyping was determined by PCR and SNaPshot sequencing. To determine whether the four SNPs account for risks in AD in Chinese population, multivariate logistic regression models were performed. Stratified analysis was performed based on gender and age of onset of AD, separately. Statistical significance was set at 0.0125 (0.05/4) based on Bonferroni correction.

**Findings:** *DHCR7* rs12785878 T>C was found to be significantly associated with an increased risk of early-onset Alzheimer's disease (EOAD) (*n* = 300, risk allele C, adjusted OR = 1.542, adjusted 95% CI = 1.176–2.023, *p* = 0.002). There was no statistical significance of the other three SNPs between the two groups.

**Interpretation:** Our results suggested that *DHCR7* rs12785878 T>C might be associated with an increased risk of EOAD in the Chinese population, while other polymorphisms related to vitamin D insufficiency might not be. However, due to the limited data in this study, replication studies in a larger sample are required.

## Introduction

Vitamin D, a steroid hormone, is crucial for maintaining musculoskeletal health and mineral metabolism. Its insufficiency has been linked to several skeletal or extra-skeletal disorders, including bone fracture, osteoporosis, diabetes, cancer, and cardiovascular disease (Martinez et al., 1996; Wang et al., 2008; Zipitis and Akobeng, 2008; Rizzoli et al., 2014). Recently, large-scale cohort studies and cross-sectional studies suggested that a decreased 25-hydroxyvitamin D [25(OH)D] level is associated with an increased risk of Alzheimer's disease (AD) (Balion et al., 2012; Afzal et al., 2014; Littlejohns et al., 2014). AD is the most common neurodegenerative disease characterized by progressive impairments in cognition, memory, language, behavior, and motor functions (Reitz et al., 2011). The pathogenetic hallmarks of AD are cerebral extracellular amyloid plaques and intracellular neurofibrillary tangles followed by oxidative damage and inflammation, leading to localized synaptic failure, and neuronal loss (Querfurth and LaFerla, 2010). Vitamin D status has shown links with AD tightly. Several longitudinal studies have observed an association of reduced plasma 25(OH)D with an increased risk of AD (Afzal et al., 2014; Littlejohns et al., 2014). Other researchers found vitamin D supplementation could improve cognitive function in elderly patients with AD (Chaves et al., 2014; Jia et al., 2019). It has been proved that vitamin D increases the phagocytic clearance of amyloid plaques and reduces amyloid-induced cytotoxicity and apoptosis (Masoumi et al., 2009; Dursun et al., 2011; Mizwicki et al., 2012). Furthermore, the hormonally active form of vitamin D−1,25-dihydroxy vitamin D [1,25-(OH)2-vitD]—has been shown to improve intracellular Ca^2+^ homeostasis dysregulated in AD (Brewer et al., 2001, 2006; Gezen-Ak et al., 2011) and to upregulate neurotrophic factors (Saporito et al., 1994; Musiol and Feldman, 1997; Gezen-Ak et al., 2011).

However, reverse causation may also exist, as the onset of dementia may limit outdoor physical activity, reduce actual sunlight exposure of patients, and subsequently cause vitamin D deficiency. Therefore, some confounding factors may influence the accuracy of the results. In some studies, the researchers used Mendelian randomization (MR), a method that minimizes bias due to confounding or reverse causation (Smith and Ebrahim, 2003), to assess the contribution of 25(OH)D in the occurrence of AD (Mokry et al., 2016). They found four single-nucleotide polymorphisms (SNPs) related to vitamin D insufficiency [described 2.44% of the variance in 25(OH)D], including *GC* rs2282679 A>C, *CYP2R1* rs10741657 T>C, *DHCR7* rs12785878 T>C, and *CYP24A1* rs6013897 T>A, were associated with an increased risk of developing AD [data from International Genomics of Alzheimer's Project (IGAP)] (Wang et al., 2010; Lambert et al., 2013), and all these four SNPs together can increase the odds of AD by 25%. This result provides evidence supporting the decrease of 25(OH)D as a causal risk factor for AD. To further test whether these SNPs associated with vitamin D insufficiency increase the risk of AD in the Chinese population, we conducted a case-control study to investigate the possible association between these four SNPs and AD in a Chinese cohort.

## Materials and Methods

### Participants

This study recruited 676 AD patients (mean age at onset was 69.52 ± 10.90 years, male: 39.2%) from mainland China. The diagnoses of probable or possible AD according to the NINCDS-ADRDA criteria (Dubois et al., 2007) were made by two or more experienced neurologists in Xiangya Hospital, Central South University. Early-onset Alzheimer's disease (EOAD) was defined as AD with an age at onset <65 years (300 subjects). Late-onset Alzheimer's disease (LOAD) was defined as AD with an age at onset more than 65 years (376 subjects). Five hundred and fifty-one healthy unrelated Chinese individuals (mean age was 67.73 ± 6.02 years, male: 44.8%) without a history of neurodegenerative diseases were recruited from the Xiangya Wellness Center as a control group. Neurocognitive assessment was performed on all AD patients, including Mini-Mental State Examination (MMSE), Clinical Dementia Rating Scale (CDR), and Activity of Daily Living Scale (ADL). MMSE was also conducted with healthy controls in this study. Informed consent for participation in the study was obtained from all subjects. This study was approved by the Ethical Review Board at Xiangya Hospital of Central South University in China.

### DNA Isolation and Genotyping Methods

Genomic DNA was extracted from peripheral blood leukocytes from all patients and controls. The quality and quantity of DNA were assessed with a fluorometer. The genotype was detected by SNaPshot, a single-base extension (SBE) assay, using ABI SNaPshot Multiplex Kit and 3730xl DNA analyzer (Applied Biosystems). Positive controls and negative controls are set for each sequencing test. All sequencing results are interpreted by two researchers using Peak Scanner-V2.0 software separately. For quality control, we randomly selected about 5% of all samples for Sanger sequencing testing, and the coincidence rate was 100%. Primers and amplification conditions required in polymerase chain reaction (PCR) are listed in Supplementary Tables 1, 2.

### Statistical Analysis

The chi-squared test was used to test for the Hardy–Weinberg equilibrium (HWE) of the genotypes. A *p*-value < 0.05 using a two-tailed test was considered statistically significant. To estimate the strength of the association between the SNPs and AD risk, multivariate logistic regression models with gender and age as covariates were performed, and the correlation strength was presented with odds ratio (OR) and 95% confidence interval (CI). For statistical significance in the association analysis, we used the Bonferroni correction to adjust the *p*-value from 0.05 to (0.05 / *k*), where *k* is the number of SNPs tested in each independent association test (*k* = 4 in our study). Therefore, a *p*-value of < 0.0125 demonstrates a significant association in statistics. Stratified analysis was performed based on gender and age of onset of AD, separately. Statistical analysis was carried out using SPSS26.0 software.

## Results

The basic information of all 1,127 subjects (676 patients and 551 healthy controls) are shown in Table 1. The genotype frequencies of the four SNPs in all controls followed HWE (*p* > 0.05). There was no statistical significance of these four SNPs in genotype or allele frequencies between the two groups (Table 2). Stratification analysis showed *CYP2R1* rs10741657 T>C was associated with decreased risk of male AD patients (*p* = 0.007, OR = 0.707); however, after being adjusted for age, the adjusted *p*-value was > 0.0125 (Table 3). We also found *DHCR7* rs12785878 T>C to be significantly associated with an increased risk of EOAD (*p* = 0.002, OR = 1.544, 95% CI = 1.177–2.025) (Table 4), and after being adjusted for sex, the difference was still significant (*p* = 0.002, adjusted OR = 1.542, adjusted 95% CI = 1.176–2.023).

**Table 1:** Demographic information of AD patients and controls^a^.

	**AD patients**	**Heathy controls**
Numbers	676	551
Age of onset/Age (years)	69.52 ± 10.90	67.73 ± 6.02
**Gender**		
Male	265	247
Female	441	304
MMSE	11.72 ± 8.55	27.98 ± 1.23

*AD, Alzheimer's disease; MMSE, Mini-mental State Examination*.

a *Data are reported as mean ± SD*.

**Table 2:** Vitamin D related gene polymorphisms in AD patients and controls.

**Genotype**	**AD**	**Control**	**Crude OR (95% CI)**	***p***	**Adjusted OR (95% CI)^b^**	***p*^b^**
**rs2282679A>C**						
AA/AC/CC	341/279/56	245/250/56	NA	0.099^a^	NA	NA
Additive			0.829 (0.697–0.987)	0.035	0.827 (0.694–0.985)	0.034
Dominant (AA/AC+CC)	341/335	245/306	0.787 (0.628-0.986)	0.037	0.780 (0.621–0.980)	0.033
Recessive (AA+AC/CC)	620/56	295/56	0.798 (0.541–1.178)	0.256	0.806 (0.545–1.193)	0.282
**rs10741657T>C**						
TT/TC/CC	100/290/286	69/228/254	NA	0.318^a^	NA	NA
Additive			0.882 (0.751–1.038)	0.130	0.888 (0.754–1.046)	0.154
Dominant (TT/TC+CC)	100/576	69/482	0.825 (0.593–1.147)	0.252	0.830 (0.596–1.158)	0.273
Recessive (TT+TC/CC)	390/286	297/254	0.857 (0.684–1.076)	0.184	0.865 (0.688–1.087)	0.214
**rs12785878T>G**						
TT/TG/GG	134/335/207	127/271/153	NA	0.311^a^	NA	NA
Additive			1.130 (0.963–1.325)	0.134	1.109 (0.944–1.303)	0.208
Dominant (TT/TG+GG)	134/542	127/424	1.212 (0.921–1.593)	0.170	1.162 (0.881–1.532)	0.289
Recessive (TT+TG/GG)	469/207	398/153	1.148 (0.896–1.471)	0.275	1.137 (0.885–1.460)	0.316
**rs6013897T>A**						
TT/TA/AA	471/185/20	370/167/14	NA	0.499^a^	NA	NA
Additive			0.928 (0.751–1.147)	0.489	0.923 (0.745–1.143)	0.462
Dominant (TT/TA+AA)	471/205	370/181	0.890 (0.699–1.133)	0.344	0.882 (0.691–1.126)	0.313
Recessive (TT+TA/AA)	656/20	537/14	1.169 (0.585–2.337)	0.658	1.186 (0.590–2.383)	0.632

*AD, Alzheimer's disease; OR, odds ratio; CI, confidence interval*.

a *Chi-Square test for genotype distributions between AD patients and controls*.

b *Adjusted for age and sex*.

**Table 3:** Vitamin D related gene polymorphisms in male/female AD patients and controls.

**Genotype**	**AD**	**Control**	**Crude OR**	**p**	**adjusted OR**	**p^a^**
			**(95% CI)**		**(95% CI)^**a**^**	
**rs2282679A>C**	AA/AC/CC					
Male	145/99/21	110/107/30	0.719 (0.551-0.936)	0.014	0.725(0.555-0.947)	0.018
Female	196/180/35	135/143/26	0.922(0.730-1.163)	0.491	0.917(0.726-1.159)	0.469
**rs10741657T>C**	TT/TC/CC					
Male	40/126/99	30/91/126	0.707 (0.549-0.911)	0.007	0.726(0.562-0.938)	0.014
Female	60/164/187	99/301/315	1.034 (0.836- 1.278)	0.759	1.027(0.830-1.271)	0.806
**rs12785878T>G**	TT/TG/GG				
Male	54/128/83	58/117/72	1.108 (0.870-1.411)	0.406	1.101(0.862-1.405)	0.441
Female	80/207/124	69/154/81	1.148(0.929-1.420)	0.202	1.118(0.903-1.385)	0.306
**rs6013897T>A**	TT/TA/AA					
Male	188/72/5	169/71/7	0.878 (0.628-1.228)	0.448	0.873 (0.622-1.224)	0.431
Female	471/205	201/96/7	0.953 (0.724-1.255)	0.733	0.957(0.726-1.262)	0.755

*AD, Alzheimer's disease; OR, odds ratio; CI, confidence interval*.

a *Adjusted for age*.

**Table 4:** Vitamin D related gene polymorphisms in EOAD/LOAD patients and controls.

**Genotype**	**AD**	**Control**	**Crude OR**	***p***	**Adjusted OR**	***p^a^***
			**(95% CI)**	**(95% CI)^b^**		
**rs2282679A>C**	AA/AC/CC					
Age <65	150/125/25	80/90/15	0.851 (0.851–1.135)	0.272	0.845 (0.633–1.129)	0.254
Age ≥ 65	191/154/31	165/160/41	0.817 (0.655–1.018)	0.072	0.822 (0.659–1.026)	0.082
**rs10741657T>C**	TT/TC/CC					
Age <65	47/130/123	17/83/85	0.783 (0.597–1.026)	0.076	0.779(0.593–1.022)	0.071
Age ≥ 65	53/160/163	52/145/169	0.947 (0.772–1.162)	0.600	0.956 (0.778–1.174)	0.666
**rs12785878T>G**	TT/TG/GG					
Age <65	42/155/103	46/93/46	1.544 (1.177–2.025)	0.002	1.542 (1.176–2.023)	0.002
Age ≥ 65	92/180/104	81/178/107	0.927 (0.758–1.133)	0.458	0.931 (0.761–1.138)	0.484
**rs6013897T>A**	TT/TA/AA					
Age <65	201/91/8	118/61/6	0.879 (0.627–1.231)	0.452	0.877 (0.626–1.229)	0.447
Age ≥ 65	270/94/12	252/106/8	0.931 (0.707–1.226)	0.611	0.924 (0.700–1.218)	0.573

*EOAD, Early-onset Alzheimer's disease; LOAD, Late-onset Alzheimer's disease; OR, odds ratio; CI, confidence interval*.

a *Adjusted for sex*.

## Discussion

In this study, we investigated the association of SNPs related to vitamin D insufficiency and the risk of AD in a cohort of Chinese subjects with AD and healthy controls. No statistically significant differences were observed in these SNPs between cases and controls. However, stratification analysis found the *DHCR7* rs12785878 T>C involved in the synthesis of vitamin D to be associated with the risk of EOAD, providing supporting evidence for the association between vitamin D insufficiency and EOAD.

Despite the detailed information about molecular pathogenic events that research has provided, little is known about the cause of AD, and no curative treatments are available. Many factors could be involved in the incidence of AD, like vitamin D insufficiency, which previous studies have found to be associated with an increased risk of AD. This is an important discovery since vitamin D repletion can be achieved safely and inexpensively through supplementation, which might reduce the occurrence of AD. However, whether vitamin D insufficiency is the cause or the result of AD remains unclear. One of the main reasons is that observational studies are susceptible to confounding factors. For example, the actual sunlight exposure of patients could not be measured accurately, and their lifestyle changes over time. Genetic background can be an entry point to solve this issue since it's not affected by factors like behavior and lifestyle that may confound observational studies.

The four SNPs associated with vitamin D insufficiency were confirmed to be related to the risk of AD, and all of these four SNPs map to genes within the vitamin D metabolic pathway (Mokry et al., 2016). *GC* rs2282679 A>C and *CYP24A1* rs6013897 T>A are associated with the metabolism of vitamin D, while *DHCR7* rs12785878 T>C and *CYP2R1* rs10741657 T>C are related to the synthesis of vitamin D. Additionally, *GC* rs2282679 A>C and *DHCR7* rs12785878 T>C may also have pleiotropic effects. *GC* encodes vitamin D-binding protein (DBP), and it is responsible for transporting most of 25(OH)D in the body (Bikle et al., 1985; White and Cooke, 2000; Verboven et al., 2002). The level of DBP has been found to increase in cerebrospinal fluid in AD patients (Zhang et al., 2008), and it can also interact with Aβ and suppress Aβ-mediated pathology (Moon et al., 2013). A recent MR study found *GC* rs2282679 A>C variant was not only associated with 25(OH)D levels but also associated with DBP levels, which indicated that *GC* rs2282679 A>C might be a pleiotropic variant (Zhang et al., 2020). *DHCR7* encodes an enzyme responsible for transforming 7-dehydrocholesterol to cholesterol, and *DHCR7* rs12785878 T>C was found to be associated with cholesterol according to a study in the Global Lipids Genetics Consortium (Willer et al., 2013). A recent analysis provided strong evidence indicating total cholesterol, low-density lipoprotein cholesterol, and high-density lipoprotein cholesterol levels as causal susceptibility factors for AD (Ostergaard et al., 2015). This provides possible evidence that this SNP may have other effects on AD. However, proofs are insufficient whether this SNP is associated with other factors that can increase the risk of AD.

Our results showed that *DHCR7* rs12785878 T>C is associated with increased risk of EOAD rather than the more common LOAD, indicating that genetic background has a more significant impact on EOAD. We did not find significant differences between cases and controls on the other three SNPs. There are several possible explanations for our negative results. Firstly, the previous study did not include the Chinese population, which means the results based on these data sources might not apply to the Chinese community even though they have included another Asian group like Indian. Since the ethnic or geographic origin of subjects can affect the frequency of SNP, this can partly explain why positive results were not replicated in our Chinese cohort. Secondly, considering our sample size is relatively small for association studies, more samples should be included to provide more substantial evidence. Although there is no strong connection between the other three SNPs associated with vitamin D insufficiency and risk of AD in our study, using genotype or allele frequency to measure the role of a risk factor in disease etiology is still useful. Since these four SNPs only explain little heritability of vitamin D, further investigation of other variations that might be associated with vitamin D and MR analysis between genetically decreased vitamin D and risk of AD in the Chinese population are needed.

## Conclusion

In conclusion, our results suggest that *DHCR7* rs12785878 T>C might be associated with risk of EOAD in the Chinese population, while other SNPs related to vitamin D insufficiency might not be. However, due to the limited data in this study, replication studies in larger samples are required.

## Data Availability Statement

The original contributions presented in the study are included in the article/Supplementary Material, further inquiries can be directed to the corresponding author/s.

## Ethics Statement

The studies involving human participants were reviewed and approved by the Ethical Review Board at Xiangya Hospital of Central South University in China. The patients/participants provided their written informed consent to participate in this study.

## Author Contributions

XLiu, LS, PW, and ZL participated in the design of the study, statistical analysis, and manuscript drafting. HW and JP collected the samples and performed experiments. BJ, LS, and ZL instructed and supervised the whole study. BJ and XLia helped to draft the manuscript and in the statistical analysis. All authors read and approved the final manuscript.

## Conflict of Interest

The authors declare that the research was conducted in the absence of any commercial or financial relationships that could be construed as a potential conflict of interest.
