# Supplementary material for: DHCR7 rs12785878 T>C Polymorphism Is Associated With an Increased Risk of Early Onset of Alzheimer's Disease in Chinese Population
Source: Front Genet. 2021 Feb 22;12:583695. doi: 10.3389/fgene.2021.583695 (PMC7938861; doi:10.3389/fgene.2021.583695)
Supplement: Supplementary file 1 [file Data_Sheet_1.docx]

**Supplementary Table 1** The primers and conditions for polymerase chain reaction

| SNPs | Primers | Conditions |
| --- | --- | --- |
| rs2282679 | F 5' TCTCCATCTTCTGACCTTGTGAT 3'  R 5' CACTGTGAGCCAATTCATTTATGTTT 3' | 95℃ 5min - {94℃ 15s - 55℃ 15s - 72℃ 30s}35cycles - 72℃ 3min - 4℃, TaKaRa Taq |
| rs10741657 | F 5' ATGAGAGGGAAGAGCAATGA 3'  R 5' ACAACAGTAGTAGGAAACAAATACA 3' |  |
| rs12785878 | F 5' GCCAAGGGATCTAGGGTTCT 3'  R 5' AGCAGACAGGACATGAGGAT 3' |  |
| rs6013897 | F 5' GCTCTGCCACTTACCTACT 3'  R 5' ACATGGATCTCAACAGGATTATATG 3' |  |

| SNPs | Primers | Conditions |
| --- | --- | --- |
| rs2282679 | ctAGCAAATCTCTGTCTCTTAATTATCTCACA | 96℃ 1min - {96℃ 10s - 52℃5s - 60℃ 30s}30cycles - 4℃, Snapshot Multiplex Kit |
| rs10741657 | AATAAGACTTTCCTTGACAGCCCT |  |
| rs12785878 | ctgactgactgaGGCTGTCTGATATCACAAAGCTTC |  |
| rs6013897 | ctgactCTGTAAAAGGGGGATAATGAAAGTACCTACTTCAG |  |

**Supplementary Table 2** The primers and conditions for extension reaction
